# The Economics of an Admissions Holding Unit

**DOI:** 10.5811/westjem.2017.4.32740

**Published:** 2017-05-01

**Authors:** Kraftin E. Schreyer, Richard Martin

**Affiliations:** Temple University Hospital, Department of Emergency Medicine, Philadelphia, Pennsylvania

## Abstract

**Introduction:**

With increasing attention to the actual cost of delivering care, return-on-investment calculations take on new significance. Boarded patients in the emergency department (ED) are harmful to clinical care and have significant financial opportunity costs. We hypothesize that investment in an admissions holding unit for admitted ED patients not only captures opportunity cost but also significantly lowers direct cost of care.

**Methods:**

This was a three-phase study at a busy urban teaching center with significant walkout rate. We first determined the true cost of maintaining a staffed ED bed for one patient-hour and compared it to alternative settings. The opportunity cost for patients leaving without being seen was then conservatively estimated. Lastly, a convenience sample of admitted patients boarding in the ED was observed continuously from one hour after decision-to-admit until physical departure from the ED to capture a record of every interaction with a nurse or physician.

**Results:**

Personnel costs per patient bed-hour were $58.20 for the ED, $24.80 for an inpatient floor, $19.20 for the inpatient observation unit, and $10.40 for an admissions holding area. An eight-bed holding unit operating at practical capacity would free 57.4 hours of bed space in the ED and allow treatment of 20 additional patients. This could yield increased revenues of $27,796 per day and capture opportunity cost of $6.09 million over 219 days, in return for extra staffing costs of $218,650. Analysis of resources used for boarded patients was determined by continuous observation of a convenience sample of ED-boarded patients, which found near-zero interactions with both nursing and physicians during the boarding interval.

**Conclusion:**

Resource expense per ED bed-hour is more than twice that in non-critical care inpatient units. Despite the high cost of available resources, boarded non-critical patients receive virtually no nursing or physician attention. An admissions holding unit is remarkably effective in avoiding the mismatch of the low-needs patients in high-cost care venues. Return on investment is enormous, but this assumes existing clinical space for this unit.

## INTRODUCTION

Boarding is recognized nationwide to be a severe problem in emergency departments (ED). Boarding prevents incoming patients from being treated, leads to increased left without being seen rates, and increases the rate of patients leaving against medical advice, a route taken by some patients frustrated with long wait times.^4^,^5^,^6^ ED visits have exponentially increased, already reaching 130.4 million in 2013.^20^ Concurrently, available hospitals, EDs associated with hospitals, and inpatient hospital beds have all decreased.^1^,^2^,^3^,^4^,^5^ By 2009, more than 90% of ED providers reported that they are operating at full ED occupancy on a consistent basis.^6^ Consequently, the United States has experienced a worsening crisis of ED crowding.^1^,^2^,^3^,^4^ Crowding, defined in a 2006 ACEP policy, occurs “when the identified need for emergency services exceeds available resources for patient care in the ED, hospital or both.”^3^ On average, patients wait almost three hours more for an inpatient bed in crowded EDs as compared to those that are not constricted by crowding, according to the Joint Commission (JCAHO).^9^ Crowding correlates with undesirable consequences, including delays in definitive treatment, increased mortality in the critically ill, and increased rates of complications leading to poorer patient outcomes.^2^,^3^,^4^,^7^ Crowding and the consequent ED boarding not only impact patient mortality and morbidity through treatment delays, but may as well have financial implications for the both the ED and the hospital by increasing hospital length of stay (LOS).^4^,^5^,^6^,^10^, ^11^ Multiple surveys show ED providers consistently ranking ED crowding as their most important patient safety concern.^9^ JCAHO identifies over one half of all “sentinel events” in cases leading to morbidity and mortality to be the result of delays in treatment in hospitals. One third of such events could have been attributed to crowding.^8^ A study by Bernstein et al. demonstrated that crowding compromises at least two of the six domains of the Institute of Medicine: safety and timeliness.^5^ Moreover, crowding has also been shown to increase provider frustration, patient and family dissatisfaction, and prolonged pain and suffering of patients.^3^,^6^,^8^

For hospitals operating at nearly full capacity, a bottleneck in output from the ED develops. Without available inpatient beds, the ED has nowhere to offload admitted patients. The lack of available inpatient beds is compounded in some hospitals by lack of flexibility between services that do not accept patients on their service in certain areas of the hospital and delays in room turnover or patient transport.^4^ Unlike inpatient units, which accept patients until beds are filled and then stop, the ED cannot close the door. EDs, some already operating at or above capacity, are forced to board patients in less-than-ideal treatment areas, such as hallway beds.^1^,^11^ This has a negative effect on patient satisfaction, as it has been shown that patients would prefer boarding in an area with more privacy than an ED hallway.^12^

Crowding can be conceptualized as the relationship between the “need for service” and “available resources.”^3^ Several solutions have been proposed to alleviate the problem of boarding, including adding additional personnel or additional ED bed space, using observation units, ambulance diversion, and eliminating non-urgent ED referrals.^6^,^17^ However, these proposed solutions are problematic. As personnel constitute the bulk of the operating budget, adding additional personnel is not always an option. It has been shown that simply increasing the number of available ED bed space, without a concomitant increase in the number of providers, does not have a substantial effect on boarding or overall LOS.^6^,^17^

In a hospital with limited inpatient bed availability, one solution to the problem of crowding and boarding is an admissions holding unit adjacent to the ED, where patients could receive good clinical care, but at less cost. To further investigate the practicality of this concept, we conducted a three-part study focusing on true cost, opportunity cost, and post-load resource utilization.

**Population Health Research Capsule**

What do we already know about this issue?

The concept of an admissions holding unit is not new, but the actual financial impact has not previously been studied.

What was the research question?

From the hospital financial officer point of view, what is the cost-benefit of a holding unit?

What was the major finding of the study?

ED boarding keeps patients in a high-cost treatment zone while using a bare minimum of clinical services.

How does this improve population health?

A holding unit allows increased access to emergency care while yielding augmented reimbursements far in excess of operating expense.

## METHODS

### Study Design

This study was conducted at an urban academic center, with an annual volume of 76,000 patients and a 26% admission rate during the study period. The study center is a trauma center, with 55 treatment spaces in the main ED, divided into three zones of high, mid and low acuity. The admissions holding unit occupies a small space (1,015ft^2^) adjacent to the ED proper, but it is not directly staffed by emergency physicians. Pre-existing space was re-purposed to create the unit. No renovations were required. The admissions holding unit in the study hospital is different than a traditional observation unit, in which patients are admitted and cared for over a 24-hour period, by ED or inpatient physicians. Patients admitted to the hospital are under the care of the inpatient teams, who provide care regardless of whether the patient is in an ED bed, an admissions holding bed, or an inpatient bed. When inpatient beds become available, patients are moved from the admissions holding unit to their assigned bed, and the admissions holding bed is then occupied by another ED patient awaiting admission. Although additional nursing staff are needed to maintain the observation unit, no additional physician staffing is necessary.

This study was performed in three phases. The first phase focused on calculating the true cost of boarded patients in the ED. The second phase focused on calculating opportunity costs for those patients who left without being seen while other patients were boarding in the ED. The final phase focused on the care provided to boarded patients, to determine their true resource utilization.

We did not include in this analysis critically ill patients admitted to an intensive care unit, as they would not be appropriate patients for the admissions holding unit. Similarly, pediatric patients were not included, as they are seen in separate section of the ED, and if admission is merited they are transferred to a nearby pediatric hospital for admission. Moreover, the admissions holding unit is not staffed by pediatric nurses. Neither did we include patients with mental health diagnoses, as all mental health admissions are transferred to a crisis center at an affiliate hospital and do not spend significant time boarding in the main ED.

#### Phase I

The first phase determined the cost of maintaining a staffed bed in the ED for a unit of time versus alternative options. Since patients do not instantaneously leave the department at the time of disposition, we arbitrarily determined that boarding time began one hour after the admission order was placed to account for the routine logistics of admission including bed assignment, nursing report, and patient transport. Boarding time ended when the patient physically departed the ED. This allowed for calculation of the true cost of boarded patients. We obtained boarding data from ED chart time stamps. Charts were abstracted from June 2010 to May 2011.

Overhead and operating cost data were obtained from the hospital finance office. The data obtained represented the direct cost to treat, and not charges. We referenced an article in the *Harvard Business Review*, “How to Solve the Cost Crisis in Health Care,” in which authors applied the concept of time-driven activity-based costing (TDABC), which has been validated in business, to medicine (Supplement).^13^,^14^ TDABC builds on the two-stage cost attribution model of activity-based costing, in which a pool of resources is created and then subsequently assigned to costly activities, by using a time equation to directly allocate costs from resource pools to products. Resources are allocated based on capacity cost rates and process time.^14^,^15^ The authors identified cost centers along the chain of medical care including the administration process (for registration) and the clinical process (for care). Costs were allocated based on the consumption of resources over time, which led to the conclusion that the longer a resource is used, the greater its cost. The more time patients spend boarding, the greater the cost to treat.^1^

To apply this concept to the study hospital, we determined capacity costs per patient bed-hour in the ED, the inpatient floors, the observation units and the holding unit, using the following formula:

$$\text{Capacity Cost }{\text{Rate}}^{\text{time-}1}=\frac{\text{Cost of Resource}}{\text{Available Capacity of Resource}}$$

The cost of the resource included all costs attributable to that resource including salary, supervision, space and equipment, for each resource identified along the chain of care. The available capacity of the resource was the available work time for both staff and equipment. Boarding costs were determined using the following formula:

$$\text{Cost of Boarding}=\left(\frac{\text{Avg cost}}{\text{Pt }{\text{Hour}}_{\text{ED}}}-\frac{\text{Avg cost}}{\text{Pt }{\text{Hour}}_{\text{Floor}}}\right)\times \text{Boarding Time }(\text{hours})+\left(\frac{\text{Avg cost}}{\text{Pt }{\text{Hour}}_{\text{ED}}}-\frac{\text{Avg cost}}{\text{Pt }{\text{Hour}}_{\text{Obs}}}\right)\times \text{Boarding Time }(\text{hours})$$

By using the delta costs for a patient in the ED versus an inpatient or observation bed and multiplying that by the actual time spent boarding, we determined total costs of boarded patients for a time period of one year.

#### Phase II

The second phase of the study looked at opportunity costs, defined as the loss of any potential gains from alternative options when a particular option is chosen.^16^ In this scenario, we calculated opportunity costs based on the implementation of an admissions holding unit in our department. It was assumed that our admissions holding unit was an eight-bed unit operating at 12 hours/day at only 60% capacity (219 days/year). The hours of free bed space that became available by using the admissions holding unit were calculated and applied to the current hours patients spent in the ED prior to disposition, as determined by times derived from chart time stamps. We then estimated the number of new patients able to be seen. We then used the actual reimbursement per patient to calculate potential increased revenue generated from additional patient encounters.

#### Phase III

The final phase of the study focused on how much care patients actually received during the post-load time, defined as an hour after the time from disposition (physician decision-to-admit) to actual physical departure from the ED. A work-study medical student observed patients minute by minute during the post-load time and recorded all interactions that patient had with any nurse or physician. Literature search did not find previous report of such granular observations, with regard to possible mismatch between resources available and resources consumed.

## RESULTS

### Phase I

**Figure 1:**
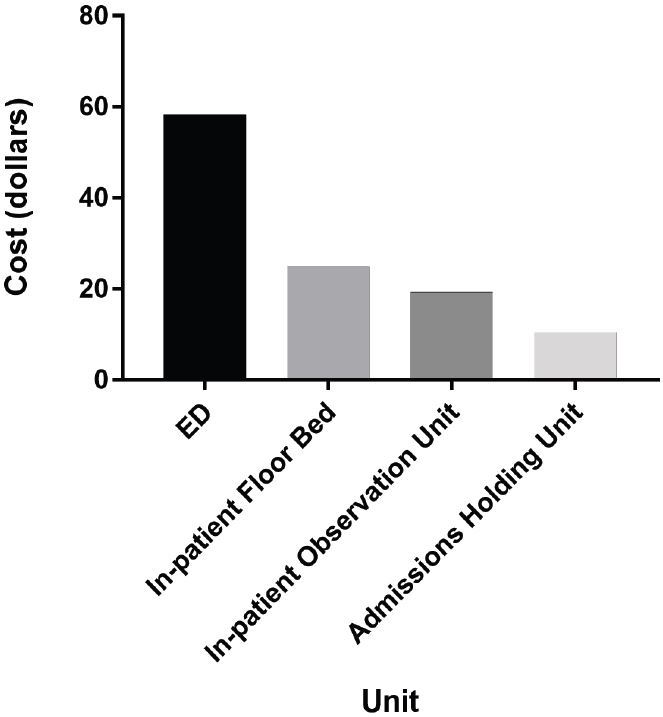
Costs per patient bed-hour by unit.

Over a typical week, patients spent anywhere from 60 minutes to 122 minutes boarding in the ED, with total time averaging 94 minutes. We calculated total boarding time over one academic year to be 32,094 hours. Costs per patient bed-hour were determined to be $58.20 in the ED, $24.80 on the inpatient floor, $19.20 in the observation unit, and $10.40 in the admissions holding unit (Figure 1). The total cost to the institution of boarded patients for one year was determined to be $877,290.

### Phase II

In the study hospital during the study time period, an average of 21.5 patients left without being seen each day. By using an admissions holding unit, it was calculated that 57.4 additional patient bed-hours per day in the department would become available, based on average turn-around times (TAT) of 3.26 hours for discharges and 6.27 hours for admissions. We performed calculations using the following formula, which assumed a one-hour adjustment for routine logistics.

$$\begin{array}{l} \text{Avg Admission TAT}-\text{Adjustment for Logistics}=\text{Hours available for new admissions}\\ \text{Avg Discharge TAT}-\text{Adjustment for Logistics}=\text{Hours available for new discharges} \end{array}$$

This would allow for four extra patients to be admitted per day and for 16 more patients to be seen, treated, and discharged. The additional patient visits would lead to increased revenue of $27,796 per day, totaling $6.09 million in the course of a year, assuming the admissions holding unit was operating at 60% capacity. We calculated revenue impact by averaging true collections of $151 for discharged patients ($100 hospital reimbursement plus $51 physician reimbursement) and $6,345 for a hospitalization. Third-party payers demonstrate varying methods of reimbursing ED charges for hospitalized patients, so these numbers represent averages, overall. Cost basis would come from increased staffing costs of $218,650 (two nurses per hour) for the number of days in operation per year, with no further overhead in locating a unit already physically equipped for patient care.

### Phase III

**Figure 2:**
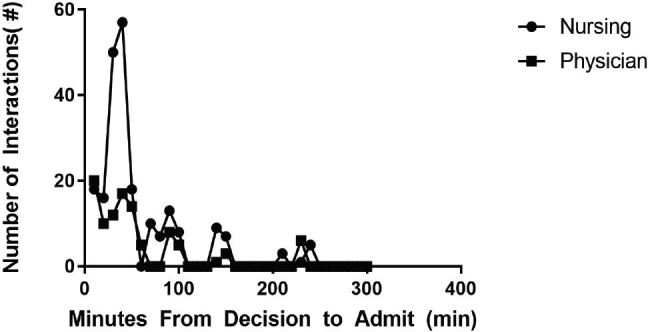
Nursing and physician care of boarded patients.

On average, admitted patients spent 2.3 hours in our ED from the time of admission to actual disposition. In that time, patients interacted with nurses an average of 2.5 times in the first 30 minutes, 1.2 times in the second 30 minutes and had near-zero interactions with nursing more than one hour after time of disposition (Figure 2). Similar data were found for interactions with physicians. In the first 30 minutes, patients interacted with physicians an average of 2.8 times. In the second 30 minutes, they interacted an average of 2.5 times. And again, there were near-zero interactions more than one hour after time of disposition. During the actual boarding time period, which does not include the first hour after time of disposition, patients had near-zero interactions with both nurses and physicians.

## DISCUSSION

We found that our patients boarded in the ED for a substantial, but variable, amount of time each day, re-demonstrating the cyclical nature of boarding noted by Handel et al. They are cared for by ED nurses, at higher staffing costs, and prevent other patients from being seen by occupying potentially available bed space. It costs twice as much to maintain the same patient in an ED bed as compared to the inpatient bed, and five times as much to keep that patient in the ED instead of a bed in the holding unit. Boarding patients have direct cost to our hospital of almost $900,000 annually.

While occupying the ED bed at a higher cost, these patients demand minimal resources. The care they receive significantly declines over time and is care that could be provided in a less costly inpatient floor bed. When no inpatient beds are available, an admissions holding unit provides a remarkably cost-effective way to provide the same amount of care, with no extra physician costs required.

Significant potential value of the admissions holding unit is the opportunity costs recouped. We showed that 20 more patients can be seen per day in an ED when the admissions holding unit is operating at 60% capacity for only half of each day. This assumption was made to reflect the practical capacity of the unit.^14^ Operating at practical capacity, the hospital is able to generate almost $28,000 more per day, and $6.09 million over only two thirds of a year.

Similar studies in a variety of hospital settings have similarly shown that by reducing boarding time by as little as 30 minutes a day, sufficient time to see an additional 8.7 to 36 patients per day is created, and there is potential for an increase in revenue by $2.7 to $3.9 million per year.^11^,^18^,^19^ Khare et al. in a computer-simulation model showed improvement in the rate at which admitted patients departed the ED and an overall improvement in LOS. They also found that simply increasing the number of available ED beds had no such impact.^17^ Huang et al. also analyzed the impact of delays of moving admitted ED patients to inpatient beds and showed that those delays impact the entirety of the hospital stay, by increasing the inpatient LOS and cost.

## LIMITATIONS

This study was conducted at a single center, and therefore, the operational factors used to calculate the economics may not be generalizable to other institutions. The economics were also calculated assuming the admissions holding unit could be placed in pre-existing space within an ED. It did not account for any cost associated with renovations that may be needed to incorporate such a unit at other institutions.

The majority of costs in this study, as obtained from the hospital finance office, were representative of staffing. It was assumed that the cost of equipment such as IVs, monitors, imaging, and medications, remained the same across the entire hospital system and would not be different in the ED versus the inpatient floors. Assumptions were also made to reflect the practical capacity of the admission holding unit, but may not be precise in determining the actual availability of staff or hours the unit was operational.

Figures for hospital and physician reimbursement used in this study, obtained from the hospital finance office, pre-date the time frame during which the study was conducted by one fiscal year. In the time between that which the figures reflect and the study period, an electronic medical record was implemented, and therefore, the actual reimbursement during the study period may have been higher.

## CONCLUSION

It costs more than twice as much to maintain the same patient in an ED bed compared to an inpatient bed and more than five times as much to keep that same patient in the ED compared to an admissions holding unit. While patients are boarding in the ED, they need and receive minimal resources. The hospital, therefore, takes a triple hit by boarding patients in the ED. Money is lost maintaining an empty inpatient bed; more money is spent keeping a patient in the resource-intensive ED setting while receiving very few resources; and, quite substantial revenue is lost by preventing patients in the waiting room from accessing care. An admissions holding unit, while not a definitive overall solution to the boarding problem, nevertheless offers a win-win strategy. Such a unit comes with cost to outfit and staff, but by providing care at less overall cost, can be seen as having great return on investment.

## Supplementary Information
