# Supplementary material for: The Economics of an Admissions Holding Unit
Source: West J Emerg Med. 2017 May 1;18(4):553–8. doi: 10.5811/westjem.2017.4.32740 (PMC5468058; doi:10.5811/westjem.2017.4.32740)
Supplement: Supplementary file 1 [file wjem-18-553-s001.docx]

**Supplement.**

**Phase I**

Applying TDABC

Cost of Boarding = (Avg cost/ Pt Hour_ED_ - Avg cost/ Pt Hour_Floor_) x Boarding Time (hours)

+

(Avg cost/ Pt Hour_ED_ - Avg cost/ Pt Hour_Obs_) x Boarding Time (hours)

*5419 = total boarding hours for patients admitted to observation units

*11077 = total boarding hours for patients admitted to med-surg units

*$58.20 = Avg cost/Pt Hour_ED_

*$19.20 = Avg cost/Pt Hour_Floor_

*$24.80 = Avg cost/Pt Hour_Obs_

$877,290 = [($58.20-$19.20) x 5419] + [($58.20-$24.80) x 11077]

**Phase II**

Hours of new Available Bed Space

Avg Admission TAT – Adjustment for Logistics = Hours available for new admissions

Avg Discharge TAT – Adjustment for Logistics = Hours available for new discharges

*6.27 hours = Avg ADM TAT

*3.26 hours = Avg DC TAT

*1 hour = Adjustment for Logistics

6.27 – 1 = 5.27 hours available for new admissions

3.26 – 1 = 2.27 hours available for new discharges

21.5 pts LWOBS/day ≈ 20 patients

4 admissions + 16 discharges = 20 patients

4 new admissions x 5.27 hours + 16 x 2.27 hours = 57.4 hours

Revenue Calculation

Discharge collections = $151 = $100 hospital reimbursement + $51 physician reimbursement

Admission collections = $6,345

*data obtained from hospital finance office*

Days Holding Unit Operational (assuming 60% capacity) - 0.60 x 365 days/yr = 219 days/yr

New discharges and admissions/day calculated above under hours of new available bed space

Revenue/day = (Discharge collections x new discharges/day x days holding unit operational)

+

(Admission collections x new admissions/day x days holding unit operational)

$27,796 = ($151 x 16 x 219) + ($6,345 x 4 x 219)

Revenue per year = Revenue/day x 219 days

$6,087,324 = $27,796 x 219

Staffing Cost

It was assumed that the cost of equipment such as IVs, monitors, imaging, and medications, remained the same across the entire hospital system and would not be different in the ED versus the inpatient floors. Therefore, the only cost included in the calculation of cost for the individual units was staffing cost.

Staffing cost = Cost/Pt Hour_Adm Holding Unit_ x Beds in Unit x Hours oper/day x Days oper/year

$218.650 = $10.40 x 8 x 12 x 219
